# Deterministic nuclear reprogramming of mammalian nuclei to a totipotency-like state by Amphibian meiotic oocytes for stem cell therapy in humans

**DOI:** 10.1242/bio.060011

**Published:** 2024-03-11

**Authors:** Ming-Hsuan Wen, Hector Barbosa Triana, Richard Butler, Hsiang-Wei Hu, Yang-Hong Dai, Nicola Lawrence, Jun-Jie Hong, Nigel Garrett, Rue Jones-Green, Emma L. Rawlins, Ziqi Dong, Magdalena J. Koziol, J. B. Gurdon

**Affiliations:** ^1^ Wellcome Trust/Cancer Research UK Gurdon Institute, Henry Wellcome Building of Cancer and Developmental Biology University of Cambridge, Cambridge CB2 1QN, UK; ^2^ Department of Zoology, University of Cambridge, Cambridge CB3 3EJ, UK; ^3^ Department of Pathology, University of Cambridge, Cambridge CB2 1QP, UK; ^4^ Department of Artificial Intelligence in Healthcare, International Academia of Biomedical Innovation Technology, Taipei 10488, Taiwan; ^5^ Department of Biomedical Technology and Device Research Laboratories, Industrial Technology Research Institute, Hsinchu 310401, Taiwan; ^6^ Department of Radiation Oncology, Tri-Service General Hospital, Taipei 114202, Taiwan; ^7^ Scientific Research Services, Phalanx Biotech Group, Hsinchu 30077, Taiwan; ^8^ Department of Physiology, Development and Neuroscience, University of Cambridge, Cambridge CB2 3DY, UK; ^9^ Chinese Institute for Brain Research, Research Unit of Medical Neurobiology, Chinese Academy of Medical Sciences Beijing 102206, China

**Keywords:** Somatic cell nuclear reprogramming, Germinal vesicle, Oocyte, Totipotency-like stem cell, Reprogramming resistant gene, Stem cell therapy

## Abstract

The ultimate aim of nuclear reprogramming is to provide stem cells or differentiated cells from unrelated cell types as a cell source for regenerative medicine. A popular route towards this is transcription factor induction, and an alternative way is an original procedure of transplanting a single somatic cell nucleus to an unfertilized egg. A third route is to transplant hundreds of cell nuclei into the germinal vesicle (GV) of a non-dividing Amphibian meiotic oocyte, which leads to the activation of silent genes in 24 h and robustly induces a totipotency-like state in almost all transplanted cells. We apply this third route for potential therapeutic use and describe a procedure by which the differentiated states of cells can be reversed so that totipotency and pluripotency gene expression are regained. Differentiated cells are exposed to GV extracts and are reprogrammed to form embryoid bodies, which shows the maintenance of stemness and could be induced to follow new directions of differentiation. We conclude that much of the reprogramming effect of eggs is already present in meiotic oocytes and does not require cell division or selection of dividing cells. Reprogrammed cells by oocytes could serve as replacements for defective adult cells in humans.

## Teaser

**Summary:** Stem cell therapy has shed light on incurable diseases. We describe a novel method for cell reprogramming and provide personalized stem cell sources for stem cell therapies.

## INTRODUCTION

The discovery that the four Yamanaka factors when overexpressed in mouse embryonic fibroblasts (MEFs), can generate embryonic stem cell-like cells (induced pluripotent stem cells, iPSCs) (Takahashi and Yamanaka, 2006) has opened the way to derive some kinds of cell-types from adult cells as a source of replacement cells in humans. In the 10 years since their result, many labs have tried to determine the route by which this transition can take place (Buganim et al., 2012; Soufi et al., 2012, 2015). There are three reasons why this has had only limited success so far. These are (i) the process is slow, usually requiring about 1-3 weeks for pluripotent cells to appear, (ii) the yield of pluripotent cells is small, i.e. only a few of the treated cells being ancestral to the derived pluripotent cells, and (iii) DNA replication and cell division of the treated cells continue during the intervening time and may be necessary for the reprogramming to take place.

We describe here a way of avoiding these limitations and hence towards identification of the earliest steps in this reprogramming process. This involves the use of oocytes (in the first meiotic prophase) as opposed to eggs (in the second meiotic metaphase) as recipients for transplanted mammalian somatic nuclei (Gurdon et al., 1958). By comparison with previous attempts to unravel the earliest stages of iPSC reprogramming, oocyte nuclear transfer completely avoids the uncertain contribution of cell division and DNA synthesis (Jullien et al., 2014; Lavagnolli et al., 2015) and the long reprogramming time (Polo et al., 2012) (3-5 days for the earliest markers of iPSC derivation, Thy1 down and SSEA1 up) is greatly shortened to 48 h at 18°C.

The requirement of cell division for nuclear reprogramming has been unclear for a long time (Pasque et al., 2010). On the one hand, somatic cell nuclear transfer to metaphase eggs and interphase zygotes shows that the former is vital for nuclear reprogramming to pluripotency due to its mitotic activity (Kang et al., 2014; Wakayama et al., 2000). On the other hand, the fusion of somatic cells with embryonic stem cells shows the early reprogramming, indicated by the activation of Oct4, is independent of DNA replication and cell division, but the later completion of reprogramming may rely on it (Do and Scholer, 2004; Han et al., 2008). The induction of pluripotency by Yamanaka factors is accompanied by several rounds of cell division for weeks, and only a small population of cells become induced pluripotent stem cells (Buganim et al., 2012; Takahashi and Yamanaka, 2006). The induction of pluripotency by Yamanaka factors (OSK), combined with the incubation of MEF in metaphase and interphase egg extracts, shows that nuclear reprogramming requires mitosis to remodel nuclei (Ganier et al., 2011). Here, we evaluated the completion of nuclear reprogramming by nuclear transfer to meiotic oocytes, which are inactive in cell division, and developed the procedure to reprogram human cells to a totipotency-like state for the potential use of cell therapy.

## RESULTS

### Deterministic nuclear reprogramming in different cell types by nuclear transfer into *Xenopus* germinal vesicle (GV) oocytes

**Fig. 1.**
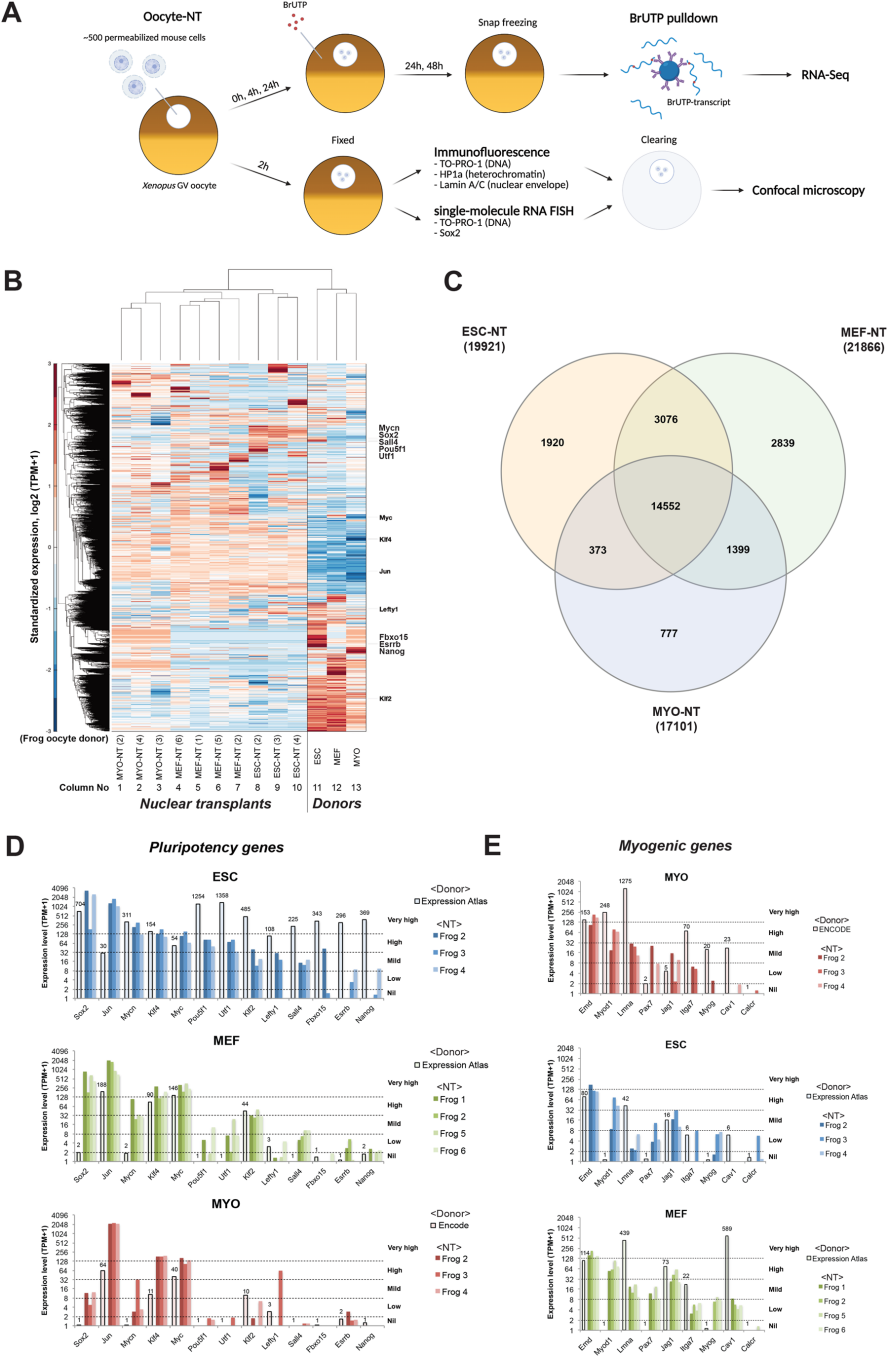
**The chromatin changes and nuclear envelope breakdown in transplanted MEFs injected in GVs of non-dividing oocytes.** (A) Schematic diagram shows the procedures of nuclear transfer to oocytes and following analyses. (B) Heatmap shows similar transcriptional changes for oocyte-induced genes in donor cell types and nuclear transplants at 48 h after nuclear transfer. Pluripotency genes are indicated at the right side of the heatmap. (C) Venn diagram shows similar gene induction by oocyte factors for three cell types at 48 h after nuclear transfer. (D,E) Bar charts show the different responses between cell types to the induction of pluripotency genes (D) and repression of myogenic genes (E) by oocyte factors at 48 h after nuclear transfer.

To observe the effect of *Xenopus* oocytes on the nuclear reprogramming of mammalian cells, we perform nuclear transfer (NT) and our procedure here is as follows (Fig. 1A). About 500 nuclei in 10 nanoliters from mouse embryonic stem cells (ESCs), MEFs or mouse myoblasts (MYOs) are injected into the GVs of oocytes, which are incubated for up to 48 h for the following analyses. The gene expression profiles of donor nuclei are from published data: Expression Atlas, EMBL-EBI, E-GEOD-27843 for ESC and MEF; for MYO, they were from ENCODE, ENCSR000AHY. In experiments for RNA-seq, six different females were used to supply recipient oocytes (Fig. 1B). We have confirmed that the results we get are not affected by differences in the source of oocytes of one female compared to those of another (Fig. 1B). Our results are therefore not affected by the source of oocytes.

We then evaluated the response of three cell types to the transplantation into oocytes. There are substantial differences in gene expression when comparing donor nuclear preparations from ESCs, MEFs, and MYOs to transplanted nuclei, analyzed by Pearson correlation coefficient (r=0.3 for ESC versus ESC-NT, r=0.07 for MEF versus MEF-NT, r=0.12 for MYO versus MYO-NT, Table S1). After nuclear transfer, from a global point of view, there is substantial similarity in the induced gene expression of the three donor nuclear types with high correlation, Pearson's r>0.78 between ESC-NT, MEF-NT, and MYO-NT (Table S1). We now ask if this similar response to transplantation extends to selecting individual genes induced by *Xenopus* oocytes in these cell types. The oocyte-inducible genes, labelled by BrUTP with TPM≥1, show a considerable, but incomplete, overlap in the three donor cell types (Fig. 1C, 14,552 in the total 24,936 oocyte-inducible genes).

Noticeably, while the inducible genes are similar in expression regardless of cell types, hierarchical clustering groups the gene expression in transplanted nuclei between one kind of donor nuclear preparation and another (Fig. 1B, compare columns 1-3 with 4-7 and 8-10). It shows the memory of donor nuclei after nuclear reprogramming by GV oocytes. Examples of individual gene expression values are shown for pluripotency genes in Fig. 1D. We see that the further donor cells are from adulthood, the higher the proportion of their genes that are activated, that is, for ESC, MEF, and MYO in that order. Many of the most strongly activated genes are present in all three donor cell types, e.g. Jun, Klf4, and Myc. If we limit our attention to individual myogenic gene transcript values, the results are similar for all three donor cell types, and most myogenic genes decrease in expression after nuclear transfer (Fig. 1E).

In conclusion, the resemblance of oocyte-induced global gene expression between distinct donor cell types shows the same directed response to GV oocyte factors and the deterministic reprogramming of transplanted nuclei after nuclear transfer.

### Early chromatin changes lead to the completion of nuclear reprogramming to a totipotency-like state by *Xenopus* oocytes within 24 h

**Fig. 2.**
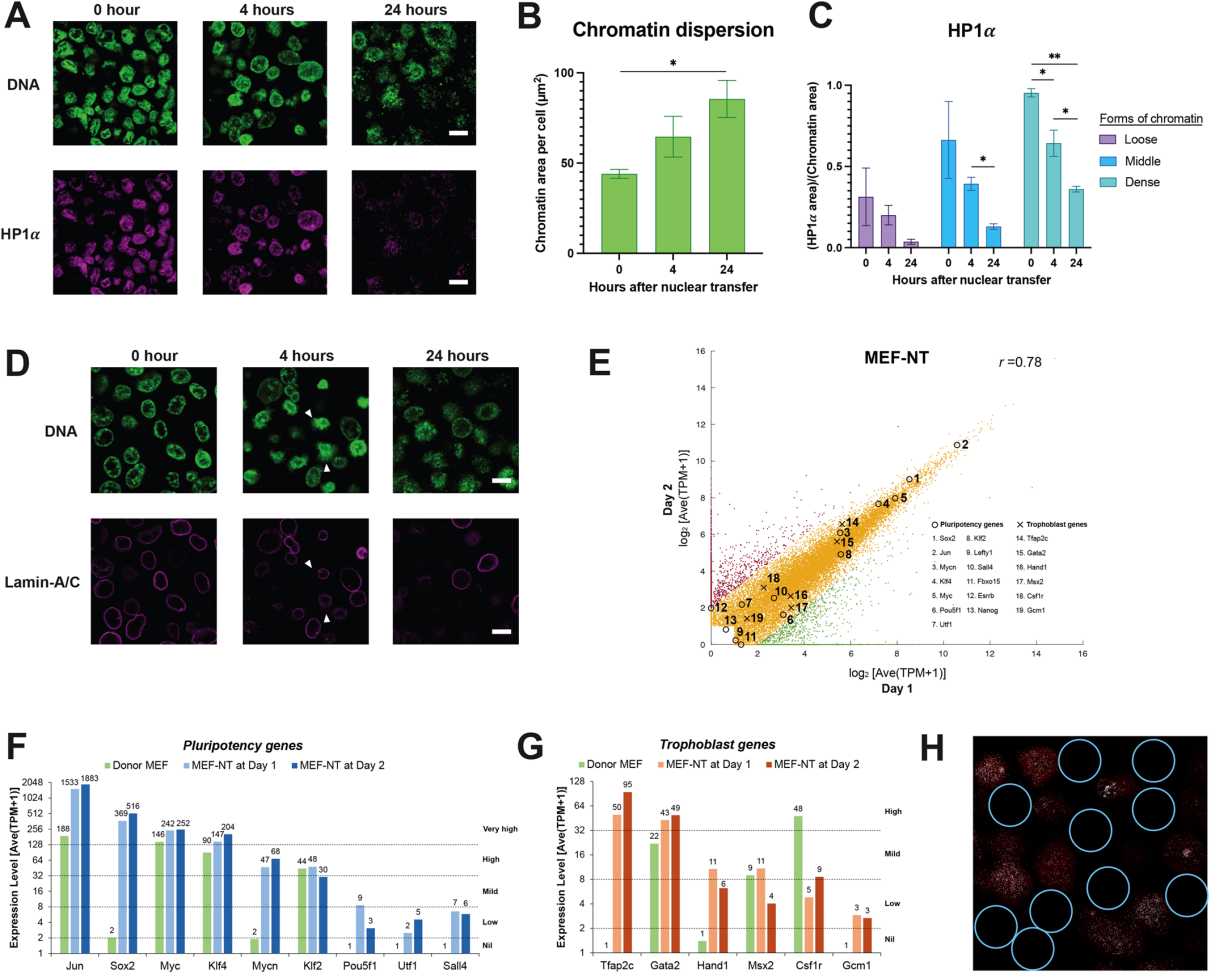
**Completion of transcriptional reprogramming to a totipotency-like state by oocytes within 24 h after nuclear transfer.** (A) Images show the dispersion of chromatin (in green) and loss of HP1α (in magenta) in MEF after nuclear transfer. Scale bars: 10 µm. (B) Bar chart shows that the chromatin in MEFs dispersed by twofold in area at 24 h after nuclear transfer. Error bars represent±SD. *P<0.05 by the Student's *t*-test, *n*=3. (C) Bar chart shows the loss of HP1α in three density levels of chromatin in MEFs from 4 h onwards. At all three time points, the more condensed the chromatin is, the more areas of chromatin occupied by HP1α. Error bars represent±SD. *P<0.05, **P<0.01 by the Student's *t*-test, *n*=3. (D) Images show the breakdown of nuclear envelope (in magenta) and burst of chromatin (in green) in nuclei of injected MEF after nuclear transfer. The breakdown of nuclear envelope where chromatin burst was observed (white arrowhead) at 4 h after nuclear transfer. Scale bars: 10 µm. (E) Dot plot shows the high correlation (r=0.78) of transcriptomes between day 1 and day 2 after MEF nuclei transplanted into *Xenopus* meiotic oocytes. (F,G) Pluripotency genes (F) and trophoblast genes (G) in transplanted MEFs were activated by oocyte factors on day 1 after nuclear transfer and remain at the same expression level on day 2. (H) Sox2 transcripts (white spots) were activated by oocyte factors in all transplanted MEFs at 24 h after nuclear transfer. The red color shows TO-PRO-1 stain for chromatin of MEFs. The areas outlined in blue show the absence of hybridized probes to the background areas between MEF nuclei.

In earlier work on nuclei injected into oocytes, we saw significant nuclear enlargement and chromatin dispersal on day 3 or 4 after nuclear transfer (Gurdon, 1976). We now ask on what time scale these effects initially occur; do these effects precede or follow changes in gene expression? To visualize chromatin changes *in situ*, we utilized optical clearing to evaluate the changes of transplanted mouse nuclei after being injected into the GV of *Xenopus* oocytes. The chromatin of transplanted MEF is marked by the DNA dye, TO-PRO-1, and heterochromatin protein 1 alpha, HP1α (Fig. 2A). Changes to chromatin structure in injected oocytes are seen in Fig. 2A-C. As early as 4 h after nuclear transfer, the chromatin changes can be seen for the chromatin dispersion and loss of HP1α attached to the nuclear envelope (Fig. 2A-C). At 24 h after MEF nuclear transfer, the chromatin of injected nuclei is about threefold dispersed in volume, judged by the twofold increase of chromatin area for each cell (Fig. 2B and Fig. S1A). The time-dependent reduction of HP1α in chromatin indicates the decondensation of heterochromatin (Fig. 2A,C and Fig. S1B). This change shows that chromatin dispersion and heterochromatin decondensation occur within 24 h of nuclear transfer (Fig. 2A-C). These chromatin conformational changes are also seen in ESC and MYO (Fig. S1C).

The drastic change of chromatin further raises the question of how the nuclear envelope of transplanted MEFs responds to the threefold dispersion of chromatin after nuclear transfer. To answer this, we used anti-Lamin-A/C antibody to mark the nuclear envelope of transplanted MEFs and observed the change of the nuclear envelope accompanying the dispersion of chromatin *in situ* (Fig. 2D). The nuclear envelope was intact at 0 h (Fig. 2D); at 4 h, the nuclear envelopes of transplanted MEFs started to break down (lower middle, white arrowhead, Fig. 2D). From the place where that took place, the chromatin contained in the donor nuclei burst out (upper middle, white arrowhead, Fig. 2D). At 24 h after nuclear transfer, most of the nuclear envelopes of transplanted MEFs disappeared and chromatin of them dispersed (Fig. 2D). Hence, the removal of HP1α and the disappearance of Lamin-A/C during oocyte reprogramming, begins with the early entry of GV factors, indicate forced disassembly of heterochromatin and is likely to reflect the robust transcriptional reprogramming induced by nuclear transfer to oocytes.

The nuclear membrane breakdown and chromatin changes are similar in nuclear transfers to eggs and oocytes. Are the transcriptional changes also similar in both types of nuclear transfers? After nuclear transfer, we measured the transcriptional changes of oocyte-induced genes in donor cell nuclei via BrUTP incorporation. We determine the newly synthesized BrUTP-transcripts in the 24-h and 48-h period after nuclear transfer and compare these BrUTP-transcripts to the published RNA-seq data for gene expression patterns of cultured cells from MEF, ESC, and MYO (Table S1).

The comparison between donor cells and nuclear transplants is summarized in Table 1. Our experiments show 29,195 genes whose transcript sequences are distinguishable for *Xenopus laevis* versus *Mus musculus* genomes (Table S2, see Materials and Methods). In 26% of the MEF genes analyzed (6197/23525, 10% for enhancement and 16% for activation), there was an increase in transcripts after mouse nuclear transfers to *Xenopus* oocytes over 24 h, and in others, a reduced expression was seen (Table 1). Together, about 40% of the MEF genes were induced to change transcription, up or down, by the combined effects of the oocyte components, and no difference between samples incubated for 24 and 48 h was seen after nuclear transfer (Table 1). These similar changes were seen in ESC and, in nearly as many, in MYO (Table 1).

**Table 1.**
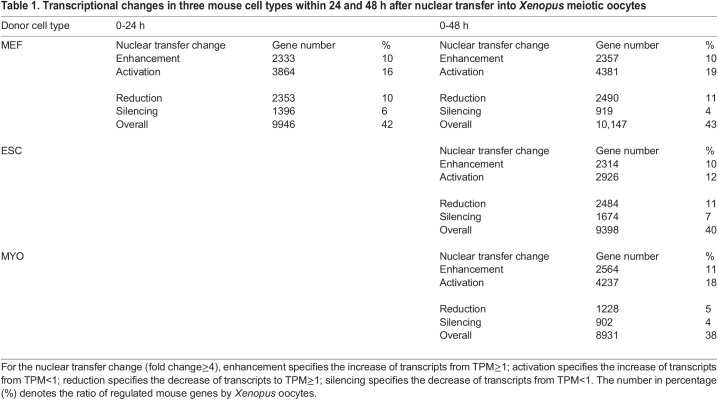
Transcriptional changes in three mouse cell types within 24 and 48 h after nuclear transfer into *Xenopus* meiotic oocytes

Global gene expression also shows that oocyte components change the gene expression pattern in donor MEF to an oocyte-induced gene expression state similar at 24 and 48 h after nuclear transfer (Fig. 2E and Fig. S1D). Most of these changes in gene expression took place in the first 24 h after nuclear transfer, as seen for MEF cells, including pluripotency genes and trophoblast genes (Fig. 2F,G). Therefore, without cell division and DNA synthesis, GV oocytes induce a totipotency-like state in transplanted MEF within 24 h after nuclear transfer, and to this extent, gene expression changes induced in eggs and oocytes are similar.

We further tested this robust nuclear reprogramming by GV oocytes via *in situ* hybridization with probes for Sox2 transcripts, and we asked what proportion of the injected MEF nuclei contain Sox2 transcripts induced after nuclear transfer. The results are shown in Fig. 2H, from which it can be seen that nearly all of the injected nuclei contain Sox2 transcripts (marked in white, Fig. 2H). Therefore, GV oocyte components effectively reprogrammed most injected nuclei (500-1000 nuclei per GV oocyte).

Overall, *Xenopus* oocytes induced drastic chromatin changes that led to the completion of nuclear reprogramming within 24 h after nuclear transfer. The robust nuclear reprogramming event triggered by GV oocyte components shows the high efficiency of nuclear reprogramming capability, and one GV oocyte can reprogram 500-1000 transplanted nuclei, as happens in nuclear transfer to egg transplants.

### Retention of gene resistance in transplanted nuclei after completion of oocyte reprogramming

**Fig. 3.**
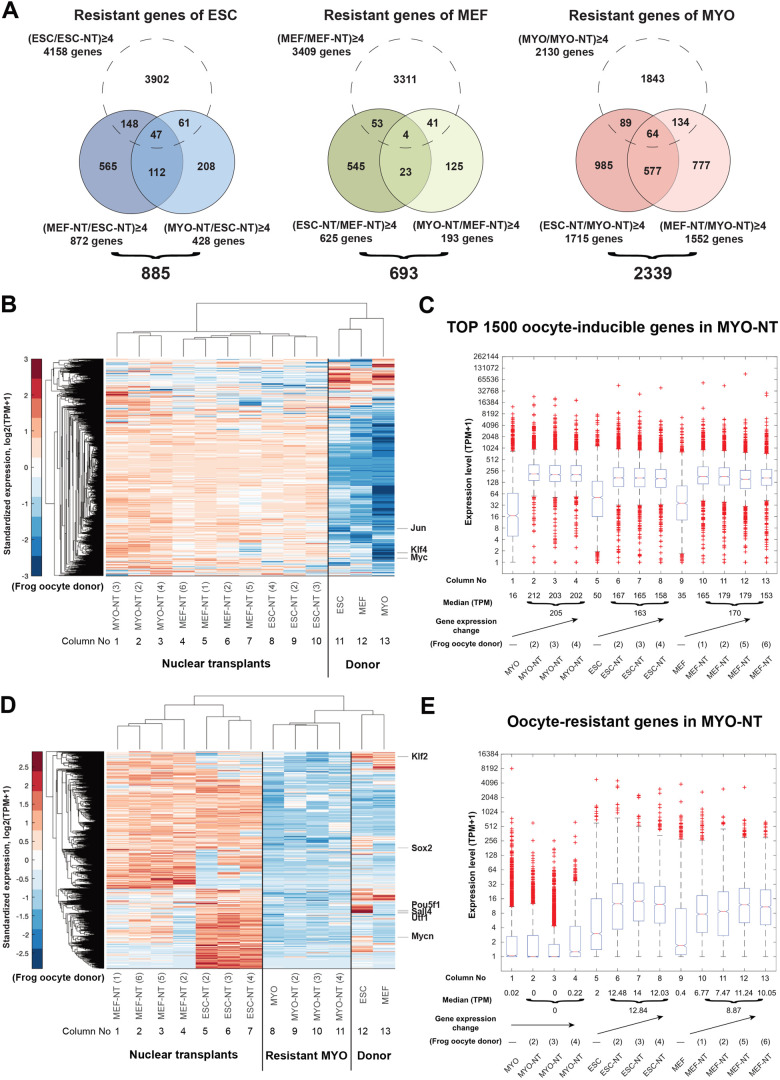
**Retention of gene resistance to oocyte reprogramming after the completion of oocyte reprogramming.** (A) Venn diagram shows the differentiated MYO nuclei are more resistant to oocyte reprogramming than embryonic ESC and MEF. Among oocyte-inducible genes, the differentially expressed genes (fold change≥ 4) indicates the gene resistance in certain cell types with lower gene expression after nuclear transfer. Considering the transcriptional activity of donor cells may affect the results, genes that are downregulated by oocytes and shown in the oocyte-resistant genes are excluded. (B) Heatmap shows three cell types respond to oocyte reprogramming similarly for the top 1500 highly expressed oocyte-induced genes in MYO-NT. The top 1500 highly expressed genes in MYO-NT includes pluripotency genes, Jun, Klf4 and Myc. (C) Boxplot shows the transcriptional increase in numbers (TPM) for the top 1500 highly expressed oocyte-induced genes in MYO-NT. (D) Heatmap shows the different response among three cell types to oocyte reprogramming for the oocyte-resistant genes in MYO-NT. Oocyte-resistant genes in MYO-NT include six pluripotency genes, Klf2, Sox2, Pou5f1, Sall4, Utf1 and Mycn. (E) Boxplot shows the transcriptional changes in numbers (TPM) for the oocyte-resistant genes in MYO-NT.

We have shown that oocytes reprogrammed transplanted nuclei and deterministically directed gene expression profiles of three cell types to a totipotency-like state. With approximately ∼25,000 genes induced by GV oocytes after nuclear transfer, gene resistance remains in three tested cell types (Table 2 and Table S3). In our results (Fig. 3A), we compared the gene expression in different cell types and quantified the resistant genes in each cell type. The MYO nuclei have a greater number of resistant genes (2339 genes) compared to ESC (885 resistant genes) and MEF (693 resistant genes). We further analyze the resistant genes in three tested cell types via gene set enrichment analysis (Table S4). In the KEGG pathway database, signaling pathways regulating pluripotency of stem cells were enriched for the resistant genes in MYO but not enriched in ESC and MEF (Table S4). The difference between resistant gene sets infers that adult cell, MYO, is more resistant to pluripotency induction than embryonic cells, ESC and MEF.

**Table 2.**
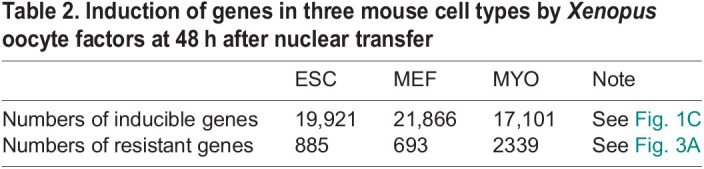
Induction of genes in three mouse cell types by *Xenopus* oocyte factors at 48 h after nuclear transfer

The distinct number and functions of resistant genes show that the susceptibility to oocyte reprogramming varied between adult cells (MYO) and embryonic cells (ESC and MEF). To examine the gene resistance to oocyte reprogramming in MYO, we list the top 1500 genes most actively expressed in MYO and the resistant genes in MYO after nuclear transfer, compared to the same gene sets in ESC and MEF. The transcriptional changes for oocyte-inducible genes in MYO are shown by heatmap and boxplot (Fig. 3B,C). The top 1500 most expressed oocyte-inducible genes in MYO nuclear transplants are up-regulated strongly by oocyte factors for all three cell types (Fig. 3B, columns 11-13 versus columns 1-10, blue to red). In the donor cells, these genes have lower expression levels (Fig. 3C, medians, 16 for MYO; 50 for ESC; 35 for MEF). After nuclear transfer, these genes are induced by oocytes to a similar median value (128≤TPM+1<256) for the three cell types with different fold changes because the initial expression levels are different between donor cell types (Fig. 3C). In contrast, the transcriptional changes of oocyte-resistant genes of MYO (Fig. 3D, columns 8 versus 9-11) differ from the same gene set for ESC and MEF, which are reprogrammed by oocyte factors more effectively (Fig. 3D, columns 1-7 and 12-13), but remain inactive in MYO after nuclear transfer, including pluripotency genes (Fig. 3D, genes listed to the right side of the heatmap). The response of resistant genes in MYO was further shown by fold-change, and the difference between adult MYO and embryonic ESC and MEF can be seen in Fig. 3E. Therefore, our results establish cell-type associated gene resistance in transplanted MYO against gene induction during oocyte reprogramming with less effect on ESC or MEF.

Overall, the effect of nuclear reprogramming by oocytes is robust, and most oocyte-inducible genes respond to oocyte factors similarly in donor cell types. The retention of gene resistance in nuclear transplants represents the susceptibility to nuclear reprogramming in each cell type and could be precisely found by the oocyte nuclear transfer experiments.

### Translational approach from nuclear transfer to *in vitro* culture for the therapeutic application to humans

**Fig. 4.**
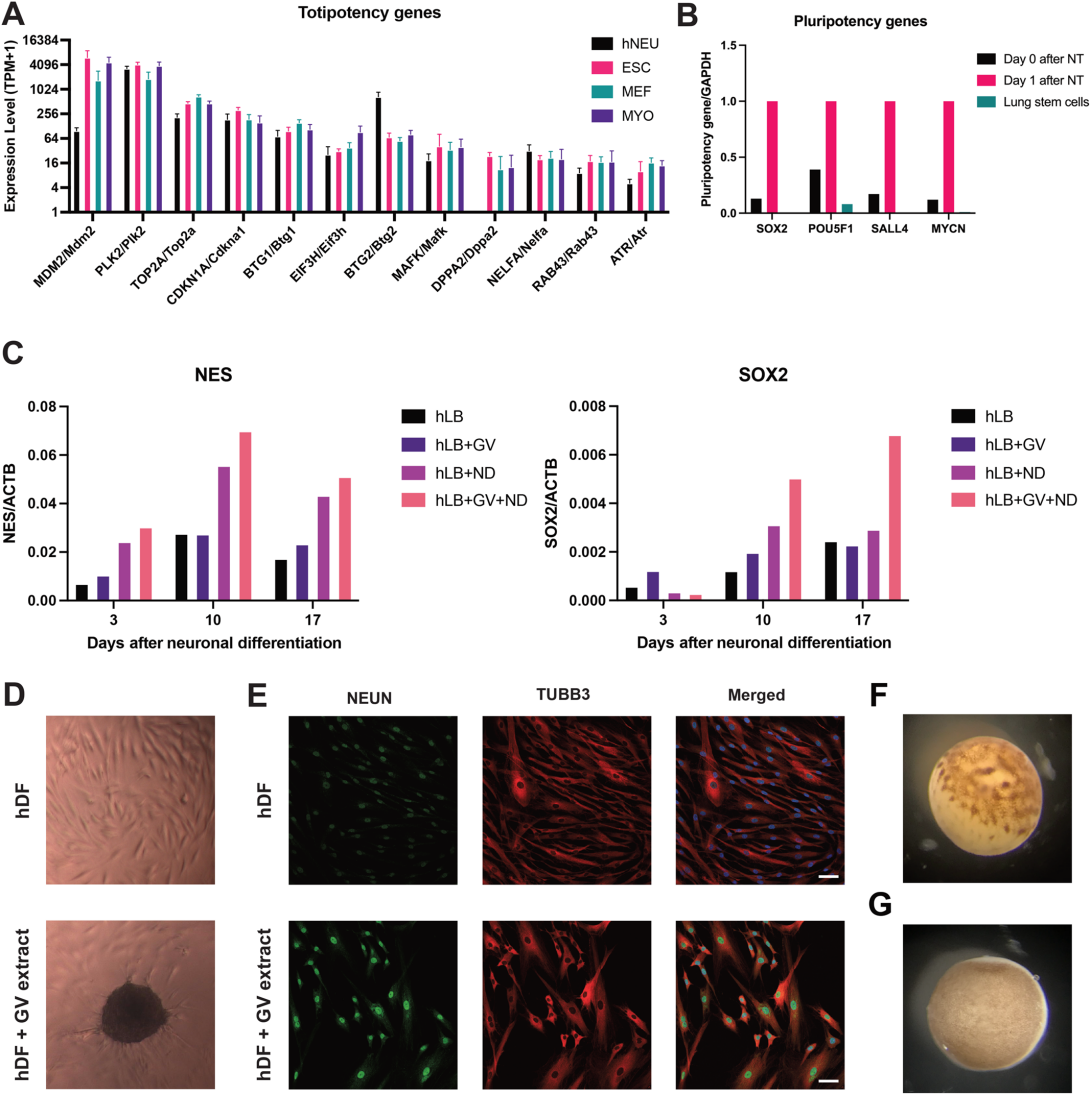
**Translational approach from nuclear transfer to *in vitro* culture.** (A) Bar chart shows the induction of totipotency genes in mouse cells (ESC, MEF, and MYO) and human cells (hNEU) on day 2 after nuclear transfer. ESC, *n*=3; MEF, *n*=4; MYO, *n*=3; hNEU, *n*=3. (B) Bar chart shows the activation of pluripotency genes in human lung stem cells after nuclear transfer. (C) Bar chart shows the increase in gene expression of the neural progenitor markers from reprogrammed hLB by GV extracts after neuronal differentiation. GV, treatment of GV extracts; ND, treatment of neuronal differentiation medium. (D) hDF formed and maintained as embryoid bodies on day 15 after GV extract treatment. The dark area in the lower figure is an embryoid body. (E) GV-extract treated hDF shows the expression of neuronal differentiation markers on day 21 after neuronal differentiation. NEUN (in green) and TUBB3 (in red) are markers for neuronal differentiation. The nuclei are stained by DAPI (in blue). Scale bar: 50 µm. (F) Image shows the unfertilized egg (metaphase II) 8 h after injecting adult human lung stem cell nuclei. (G) Image shows the oocyte in meiotic prophase I 3 days after the injection of adult human lung stem cell nuclei.

Our nuclear transfer experiments showed that transplanted mouse nuclei were reprogrammed to a totipotency-like state by *Xenopus* GV oocytes deterministically within 24 h. The cross-species interaction between mouse cell nuclei and *Xenopus* GV oocytes indicates the promising application of applying GV contents for nuclear reprogramming human cultured cells. To evaluate if the nuclear reprogramming of mouse cells can also be applied to human cells, we performed the same nuclear transfer procedures and ensured that the activation of mouse and human totipotency genes during GV oocyte reprogramming is comparable. We have demonstrated that totipotency genes are induced by *Xenopus* meiotic oocytes in both mouse cells (ESC, MEF and MYO) and human neuroblastoma cells (hNEU) to similar levels after nuclear transfer (Fig. 4A; Table S2). The induction of totipotency genes and completion of transcriptional reprogramming in both mouse and human cells direst us to test human lung stem cells, which are directly derived from human embryos. Pluripotency genes were activated successfully for human lung stem cells via nuclear transfer to oocytes (Fig. 4B), and these may be relevant to alleviating human respiratory malfunction.

We, therefore, developed our procedure to apply our oocyte GV extracts to cultured human cells and show the activation of pluripotency genes in human blood lymphoblasts (hLB). We then tested the switch of cell types by asking whether hLBs adopt the gene expression of neural cells after 3, 10, or 17 days of culture in neuronal differentiation media (Fig. 4C). This is indeed the case, as seen in Fig. 4C where hLBs are seen to express the neuronal marker genes NES and SOX2 after being treated with GV extracts and neuron differentiation medium. This change in expression is what we look for if oocyte-rejuvenated cells are to have therapeutic value.

However, the change in gene expression does not enable the suspended hLBs to be transformed into morphologically attached neuron cells. Hence, we developed our methods by using another cell type, human dermal fibroblasts (hDFs). Following the same reprogramming procedures by GV extracts, reprogrammed hDFs successfully formed embryoid bodies after 4 days in culture and maintained the function as embryonic stem cells for up to a month (Fig. 4D). We then treated the hDFs with neural differentiation factors for 3 weeks. GV-reprogrammed hDFs differentiated into neuron-like cells morphologically and expressed two neuron gene markers, NEUN and TUBB3 (Fig. 4E).

It is remarkable that *Xenopus* oocyte GV extracts can rejuvenate gene expression in mammalian nuclei, even though mammalian nuclei injected into eggs (Fig. 4F, in mitotic metaphase II) undergo very abnormal cell division and do not permit normal development. The best explanation for this is that the oocyte (Fig. 4G, in meiotic prophase I) completely lacks the components of eggs that induce cell division but already contains those components that convert oocyte chromatin into a transcriptionally active state. This active chromatin state permits gene expression but does not cause chromosome damage as induced cell division does. This is an amazing switch in the activity of oocytes as they go from meiotic to mitotic activity, and this is normally brought about by progesterone hormonal activity in the course of about 24 h.

We conclude that the incubation of human cells in *Xenopus* oocyte GV extracts mimics the reprogramming process via nuclear transfer into GV oocytes. Transcriptionally and functionally, GV-reprogrammed human cells express totipotency and pluripotency genes and maintain the stemness in the form of embryoid bodies.

## DISCUSSION

We have pursued the idea that nuclear reprogramming to a totipotent-like state by GV extracts of frog meiotic oocytes could benefit human therapy. This could certainly be the case if adult human cells of different kinds lose their normal function, as can happen with age and disease. Following an earlier report (Hansis et al., 2004), two recent papers have described the derivation of human pluripotent cells, one by Yamanaka factors and one by chemicals (Gill et al., 2022; Guan et al., 2022). Human fibroblasts can be grown from most human adult tissues. Nucleated human blood cells, such as lymphocytes and human blood stem cells, are readily obtained and turned into growing cultures, as for many other cell types. Our GV-extract procedure can reprogram These cultured cell types, and new ones can then be formed.

Here, we show that HP1α is removed during oocyte reprogramming. It is known that HP1α is crucial for the higher-order structure of constitutive heterochromatin and binds to the H3K9me3 for gene repression and silencing (Bannister et al., 2001; Machida et al., 2018). Nuclear transfer of somatic nuclei to eggs and oocytes shows the resistance of genes is marked by H3K9me3, which is alleviated by ectopic H3K9me3 demethylase Kdm4d (Jullien et al., 2017; Matoba et al., 2014). Furthermore, we show the breakdown of nuclear envelope happens during oocyte reprogramming, accompanying the disappearance of Lamin-A/C, which constitutes the nuclear lamina with other intermediate filaments. The Lamin-A/C-composed nuclear lamina is tethered to HP1-H3K9me3-marked peripheral heterochromatin and functionally linked to gene repression/silencing (Harr et al., 2015; van Steensel and Belmont, 2017).

The rapidity and extent of changes in gene expression are very pronounced during oocyte nuclear transfer, compared to what has been shown in iPSC experiments (Liu et al., 2014; Polo et al., 2012). The difference is even more significant if we allow for the fact that oocyte nuclear transfers are cultured at 18°C for amphibia compared to 37°C for mammals. We have considered that only a few injected nuclei in the oocytes may progress to the stage when pluripotency genes are expressed. This is what happens in iPSC experiments by comparison. In iPSC experiments, usually, only a small percentage of the DNA-transfected adult cells are switched to pluripotency to behave like embryos. Unequal cell division and selection when DNA-transfected cells are grown means that most DNA-transfected cells do not progress to a pluripotent, ESC-like state. Does the low percentage response also happen with nuclei-injected oocytes?

Our study used *Xenopus* meiotic oocytes and GV extracts to reprogram mouse nuclei towards totipotency and investigate the benefits of utilizing *Xenopus* meiotic oocytes to reprogram cells. Different from iPSC approach and nuclear transfer to mature eggs, nuclear transfer to meiotic oocytes does not require pioneer transcription factors or DNA synthesis/cell division. However, it uses maternal factors to increase the accessibility of chromatin to facilitate transcriptional reprogramming of hundreds of transplanted nuclei within hours. We compared the results of nuclear transfer to oocytes as described here with nuclear transfer to eggs. Are the kinds of genes activated the same by these procedures? There is certainly a massive difference in this respect. We find that nearly 40% of the mouse genes tested here underwent a gene expression change in oocyte nuclear transfers, in most cases towards a more totipotent level of gene expression (Malik and Wang, 2022; Xu et al., 2022). Some previous work has been done on the difference between nuclear reprogramming by oocytes and eggs. Alberio et al. (2005) show that the remodeling of the nuclear lamina is similar in both oocyte and egg reprogramming; however, the transcriptional activity is different (Alberio et al., 2005). A major difference between oocyte nuclear transfers and iPSC approach concerns the timing of the changes induced. Most of the changes seen in oocyte nuclear transfers occur within 24 h, and there is very little change in 48 compared to 24 h. The rapidity of changes is similar for all donor cell types used here. Oocytes show fast and efficient reprogramming competence to induce a totipotency-like state, compared to iPSC methods (Giulitti et al., 2019).

We then asked whether the components of oocytes needed for reprogramming are the same as those needed for egg nuclear transfers and for iPSC approach. We think mostly not. Most of the molecules needed for egg nuclear transfers are not yet identified but are likely to do with DNA synthesis and chromatin dispersion. There is no DNA synthesis in oocytes, but the components needed for chromatin structural changes could be similar in oocytes and eggs. These could include nucleoplasmin, DNA demethylases, etc. Lastly, we ask whether the mechanisms used for reprogramming are similar for oocytes and the other routes. We have shown here that DNA synthesis and cell selection are not required in oocytes but very well may be important in iPSC method and nuclear transfer to eggs. Other reprogramming components of eggs, including Gli1 (Maekawa et al., 2011) and Brg1 (Hansis et al., 2004), are not known to be required for reprogramming by oocytes. Previously, *Xenopus* Wave1 was shown to be essential for nuclear reprogramming in oocytes (Miyamoto et al., 2013).

Our future work will aim to identify other oocyte reprogramming components, using the antibody procedure of Clift et al. (2017). The main conclusion from the present nuclear transfer to oocyte experiments and GV extract treatment is that the most effective somatic reprogramming factors are present in the germinal vesicles of meiotic oocytes, and the completion of nuclear reprogramming is achieved within a day for oocyte-reprogrammed cells, promising for the application on stem cell therapy in humans.

## MATERIALS AND METHODS

### Isolation of oocytes from *X. laevis*

The use of female *X. laevis* for oocyte isolation is covered under the Home Office Project License PPL 70/8591. Frog husbandry and all experiments were performed according to the regulatory standards. The benign and painless procedure for obtaining oocytes from *X. laevis* is to subcutaneously inject a female frog with 0.3 g of ethyl 3-aminobenzoate methanesulfonate salt (MS222) in 0.5 ml water. The injected frog is kept on ice on its back for 15 min, after which it should be fully anaesthetized and cannot turn itself the right way around. The ovaries were removed from the anaesthetized frog. The oocytes connected with stroma tissue were torn apart as strings by forceps, exposing each oocyte to the medium. The necessary amount of ovarian material, usually 3 ml, was added to 1X MBS, collectively 12.5 ml in 50 ml conical tube [Modified Barth-HEPES Saline, 88 mM NaCl, 1 mM KCl, 2.4 mM NaHCO_3_, 0.8 mM MgSO_4_, 0.4 mM CaCl_2_, 0.33 mM Ca(NO_3_)_2_, 5 mM HEPES (pH7.8)]. The strings of oocytes were then treated with 250 µl liberase (28 U/ml in H_2_O, Roche, 5401020001) for 2 h with gentle agitation. Then the liberase was washed away from the oocytes with 1X MBS, and the stage V/VI oocytes (Dumont, 1972) with a diameter range of 1 to 1.2 mm were selected for the following experiments. The selected oocytes were placed in a Petri dish in 1X MBS at 16°C, and the follicular cell layer detached overnight. The oocytes were then stored in 1X MBS, containing 0.1% bovine serum albumin (BSA) and antibiotics. The oocytes can be kept at 16°C for several days.

### Cell culture

Mouse embryonic stem cells were cultured in G-MEM BHK-21 (Gibco, 21710-025), 20% fetal bovine serum (FBS, Gibco, 10439-024), 1000 U/ml leukemia inhibitory factor (Chemicon, ESG1107), 0.1 mM non-essential amino acids (Gibco, 11140050), 0.1 mM β-mercaptoethanol and 10 mM sodium pyruvate (Gibco, 11360070) in a gelatin-coated flask. A mouse embryonic fibroblast cell line (Pasque et al., 2011) and a mouse myoblast cell line, C2C12 (ATCC, CRL-1772), were cultured in DMEM (Sigma, D5671) with 10% FBS (ThermoFisher Scientific, 26140079). Human lung stem cells were derived from fetal lung epithelial tips and grown as long-term self-renewing organoids (Nikolic et al., 2017). Human lymphoblast cell line K-562 (Merck, 89121407-1VL) was cultured in IMDM (ThermoFisher Scientific, 31980030) with 10% FBS. Human adult dermal fibroblasts (ThermoFisher Scientific, C0135C) were cultured in medium 106 (ThermoFisher Scientific, M106500) with a low serum growth supplement kit (ThermoFisher Scientific, S003K). Human neuroblastoma cell line SH-SY5Y (ATCC, CRL-2266) was cultured in Advanced DEME/F12 (Gibco,12634010) with 10% FBS (ThermoFisher Scientific, 26140079).

### Cell permeabilization

Cells were cultured to sub-confluence, washed twice with PBS, and detached with 1X trypsin-EDTA (Gibco, 25200056) for 5 min at 37°C. Trypsin was neutralized by culture medium with BSA and centrifuged at 500 rpm for 4 min. The supernatant was discarded, and the cells were resuspended in PBS. Cells were centrifuged at 2000 rpm for 1 min, and PBS was replaced with SuNaSp solution (250 mM Sucrose, 75 mM NaCl, 0.5 mM Spermidine, 0.15 mM Spermine). Cells were centrifuged at 2000 rpm for 1 min, and the supernatant was discarded. 20 µl streptolysin O (SLO, 20,000 units/ml in PBS, containing 0.01% BSA and 5 mM DTT, Sigma-Aldrich, S5265) was added plus 100 µl SuNaSp solution for 3-6×106 cells. Cells were then resuspended by pipetting and permeabilized at 37°C in a water bath for 1 min. Cells were incubated on ice, and some cells were taken to check permeabilization efficiency (∼95-99%) under a microscope by Trypan Blue staining. SLO reaction was stopped by adding SuNaSp BSA. Cells were centrifuged at 2000 rpm for 1 min. The supernatant was discarded and resuspended with SuNaSp BSA solution for cell concentration at ∼500 nuclei/10nl. Cells in SuNaSp BSA solution were then aliquoted, snap-frozen on dry ice, and stored in a −70°C freezer.

### Nuclear transfer and cytoplasmic injection

Permeabilized cells were mixed with plasmid DNA encoding cytoplasmic membrane GFP (Halley-Stott et al., 2010). Cell suspension (9.2 nl, ∼500 cells) with 5 pg plasmid DNA was injected into the germinal vesicle of each oocyte via Drummond Nanoinjector. Two hours after nuclear transfer, 5 nl BrUTP solution (100 mM in H_2_O, Sigma-Aldrich, B7166) was injected into the cytoplasm of each oocyte. GFP-positive oocytes for oocytes were collected beyond 24 h of nuclear transfer, snap-frozen on dry ice, and stored in a −70°C freezer.

### Confocal microscopy and analysis

Immunofluorescence of HP1α and Lamin-A/C is referred to in Nguyen et al. (2019). After nuclear transfer, oocytes were fixed in low FG fixative and post-fixed with MeOH/EGTA. The oocytes were rehydrated in a sequence of 25%, 50%, 75%, and 100% TBS/MeOH and hemisected. Oocytes were bleached with bleach solution (1% H2O2, 5% formamide, 150 mM NaCl, 16 mM sodium citrate, pH 7.0 adjusted by NaOH). The transplanted nuclei were stained with anti-HP1α antibody (Alexa Fluor 647, Abcam, ab198391) or anti-Lamin-A/C antibody (Alexa Fluor 680, Santa Cruz Biotechnology, sc-376248) and then the chromatin were stained with DNA dye, TO-PRO-1 (ThermoFisher Scientific, T3602). Oocytes were cleared with Murray's clearing solution for microscopic imaging by Zeiss LSM880 Confocal Microscope. Chromatin density was quantified using a custom script for Fiji that applies a multi-level Otsu threshold with the histogram for calculation limited to values greater than or equal to the threshold from the previous iteration (https://github.com/gurdon-institute/DNA_Density/blob/main/Wen-Butler_Chromatin_Density.py). Areas of nuclei were measured by segmenting the Huang thresholded masks of TO-PRO-1 images using a recursive algorithm that watersheds sub-regions using decreasing distance map tolerance values and extracts objects that are small enough to be considered individual nuclei at each stage (https://github.com/gurdon-institute/DNA_Density/blob/main/Wen-Butler_Recursive_Watershed.py). An area overlaying the chromatin of injected nuclei is marked, and each pixel in that area is scored for signals from the TO-PRO-1 labelled chromatin. This DNA dye TO-PRO-1 was used to classify the chromatin into three density levels that are loose, middle, or dense.

### Single molecule RNA fluorescence *in situ* hybridization

After nuclear transfer, oocytes were fixed in MEMFA (100 mM MOPS, pH 7.4, 2 mM EGTA, 1 mM MgSO_4_, 3.7% formaldehyde) for 1 h at room temperature (RT) and immersed in 100% MeOH at −20°C for at least 48 h. Oocytes were rehydrated with a series of TBST/MeOH (25%, 50%, 75% and 100%), two times for each solution for at least 30 min. The oocytes were hemisected and bleached with 1% H_2_O_2_, 5% formamide and 1× SSC for 16 h. Then the oocytes were washed with TBST for 30 min twice and then immersed in Stellaris Wash Buffer A (LGC Biosearch Technologies) for 30 min at RT. The Stellaris Buffer A was then removed, and the oocytes were put in Stellaris Hybridization Buffer (LGC Biosearch Technologies) and allowed to settle for 5 min. Hybridization Buffer was then removed and oocytes were immersed in 125 nM of probe working solution of Sox2 (Stellaris RNA FISH Probes of Sox2, LGC Biosearch Technologies) at 37°C in the dark for 16 h. Following this the probe working solution was removed and the oocytes were washed with Hybridization Buffer. The chromatin was stained using 5 µM TO-PRO-1, diluted by Wash Buffer A. Oocytes were washed with TBST for 15 min twice. They were then Postfixed by 1% formaldehyde/TBST at RT for 1 h, washed with TBST for 30 min twice and dehydrated with 100% MeOH for 16 h. MeOH was removed and Murray's clearing solution was added. Oocytes were allowed to set at the bottom of tubes, and they were then imaged using Zeiss LSM880 Confocal Microscope.

### RNA extraction

Qiagen RNeasy Mini Kit (QIAGEN, 74104) was used for RNA extraction, and procedures were modified for our purpose. Briefly, oocyte samples were lysed with 900 µl RLT buffer and vortexed for 4 min at 4°C. 900 µl 70% ethanol was added and the mixture was transferred to RNeasy spin columns. The mixture was centrifuged at 10,000 rpm for 30 s and flow-through was discarded. 350 µl RW1 buffer was added, and the mixture was centrifuged at 10,000 rpm for 30 s. Flow-through was discarded and 80 µl DNase I incubation mix was added. The mixture was incubated at room temperature for 15 min, then 350 µl RW1 buffer was added, and centrifuged at 10,000 rpm for 30 s. Flow-through was discarded, 500 µl RPE buffer added, and then the mixture was centrifuged at 10,000 rpm for 2 min. RNeasy spin columns were placed in new 2 ml collection tubes and centrifuged at full speed for 1 min. The RNeasy spin columns were again placed in new 1.5 ml tubes and 50 µl RNase-free H2O was added. This was then centrifuged at 10,000 rpm for 1 min and RNA concentration was measured by Nanodrop. RNA extracts were snap-frozen on dry ice and stored at −70°C in a freezer.

### Reverse transcription and QPCR

SuperScript III Reverse Transcriptase Kit (ThermoFisher Scientific, 18080) was used for reverse transcription. SYBR Green JumpStart Taq Ready Mix (Sigma-Aldrich, S9939) was used for QPCR. Primers for pluripotency genes are listed in Halley-Stott's paper (Halley-Stott et al., 2010). 50 ng cDNA per well of a QPCR plate was used and Gapdh was used for normalization.

### Immunoprecipitation of BrUTP incorporated RNA

The protocol was adapted from a published protocol (Jullien et al., 2017). anti-BrUTP conjugated agarose beads (Santa Cruz Biotechnology, sc-32323 AC) were washed twice with Buffer I (0.5X SSPE with 0.05% Tween 20 and 0.1% polyvinylpyrrolidone) and blocked with Blocking buffer (Buffer I with 1 mg/ml RNase free BSA) at 4°C for 1.5 h. Beads solution was centrifuged at 3000 rpm for 3.5 min at 4°C and supernatant was removed. 25-50 µg RNA extract per sample was used and 2.5 µl SUPERase•In RNase Inhibitor (ThermoFisher Scientific, AM2696) was added into each sample. RNA extract was then heated at 65°C for 5 min and incubated on ice for at least 1 min and spun down. RNA was immunoprecipitated with 200 µl RIP buffer (anti-BrUTP beads in 0.5X SSPE with 0.05% Tween 20) overnight at 4°C. RNA bead mixture was washed with Low salt buffer (0.2X SSPE with 0.05% Tween 20), and with High salt buffer (0.5X SSPE with 0.05% Tween 20, and 150 mM NaCl) twice and then with TET buffer (10 mM Tris, 1 mM EDTA, pH 8 with 0.05% Tween 20). Immunoprecipitated RNA was eluted with Elution buffer (5 mM Tris, pH 7.5 with 300 mM NaCl, 1 mM EDTA, 0.1% SDS, 20 mM dithiothreitol) by incubating at room temperature for 1 min. It was then centrifuged at 3000 rpm for 4 min and the supernatant was collected. The elution step was repeated four times. Eluted RNA was extracted by phenol/chloroform extraction and ethanol precipitation. RNA extract was cleaned up with Qiagen RNeasy Plus Micro Kit (Qiagen, 74034).

### Library preparation and sequencing of RNA-seq

Ovation Single Cell RNA-seq System (NuGEN, Part No 0342) was used to prepare RNA-seq libraries from newly synthesized RNA. 10 ng of newly synthesized RNA was used for each sample preparation. Steps were followed according to the manufacturer's instructions. cDNA reverse transcribed from newly synthesized RNA was then obtained and amplified as RNA-seq libraries. RNA-seq libraries were validated by Agilent 2200 TapeStation and sequenced on Illumina HiSeq 2000 and 4000 for SE50.

### Filtering and mapping of sequencing data

Fasta files from *M. musculus* (mm10) and *X. laevis* (xla9.1) were concatenated one after the other in order to create a hybrid large *mouse-Xenopus* genome. To distinguish the *X. laevis* chromosomes in the fasta file, they were renamed as ‘xla_chr’ instead of just ‘chr’. Similarly, the gtf files containing the annotation of all transcripts from Mouse (mm10) and of all primary transcript from *X. laevis* (xla9.1) have been concatenated. FastQ files were processed with *cutadatp* (version 1.9.1, options *-q 10 -O 3*) for adapter trimming. Filtered reads were then aligned to the hybrid mouse-*Xenopus* genome with *tophat* (version v2.1.1). Transcripts were assigned to gene and counted using *htseq-count* (HTSeq-0.5.4p3). The *M. musculus* (mm10) was replaced with *H. sapiens* (hg38) in experiments with human cells transplanted into *Xenopus* oocytes.

### Gene set enrichment analysis

Functional analysis were carried out via g:Profiler (https://biit.cs.ut.ee/gprofiler/gost) for reprogramming resistant genes in ESC, MEF and MYO.

### *In vitro* reprogramming by GV extracts

Oocytes were released from ovaries of *X. laevis* (see above, Isolation of oocytes from female frogs). GVs were manually dissected in the mineral oil and the GVs were collected in a tube. GVs were snap-frozen on dry ice and stored in a freezer at −70°C. Calcium-free IMDM medium (United States Biological, I8750-08) was added and the mixture was then centrifuged at 16,100×***g*** for 10 min at 4°C. The supernatant was taken and GV extract was placed on ice, or at −20°C, for later use. Human lymphoblasts K-562 and human adult dermal fibroblasts were permeabilized by SLO in PBS (see above, Cell permeabilization). Permeabilized cells were then cultured in calcium-free IMDM medium with GV extract for 6 h and then culture medium with CaCl_2_ (final 2 mM) was added for resealing membrane. GV-extract reprogrammed cells were seeded on Matrigel-coated plates and cultured in mTeSR1 Plus medium (STEMCELL Technology, 100-0276) for up to 1 month.

### Neuronal differentiation

The neuronal differentiation protocol was modified from a previously published paper (Giulitti et al., 2019). GV-extract reprogrammed cells were seeded on Matrigel-coated plates at a density of 530 cells/mm^2^ and cultured in mTeSR1 Plus medium (STEMCELL Technology, 100-0276) for 6 days. From day 0 to 2, cells were cultured in neural medium (N2B27, 1% NEAA, 200 ng/ml L-ascorbic acid) added with 20 ng/ml bFGF (STEMCELL Technology, 78003.1) and 0.1 μM LDN 193189 (STEMCELL Technology, 72147). The cells were then cultured in neural medium supplemented with 0.1 μM LDN 193189 and 10 μM SB431542 (STEMCELL Technology, 72234) on day 3. The following 6 days, the same medium used on day 3 was supplemented with 1 μM all-trans retinoic acid (Merck, R2625) and 1 μM SAG (Merck, 566661). On day 10 to 15, the cells were cultured in neural medium supplemented with 5 μM DAPT (Merck, D5942), 4 μM SU-5402 (Merck, SML0443), 1 μM all-trans retinoic acid and 1 μM SAG. On day 16, cells were seeded on glass-bottom chamber slide and cultured in maturation medium, which contained neural medium supplemented with 20 ng/ml BDNF, 10 ng/ml GDNF, 10 ng/ml CNTF (PeproTech, 450-02, 450-10, 450-13) and 10 μM ROCK inhibitor (STEMCELL Technology, 72302). The cells were cultured in maturation medium up to day 22 and fixed for immunostaining. All the culture media were changed daily.

## Supplementary Material

10.1242/biolopen.060011_sup1 Supplementary information

Table S1.

Table S2.

Table S3.

Table S4.
