# Supplementary material for: Deterministic nuclear reprogramming of mammalian nuclei to a totipotency-like state by Amphibian meiotic oocytes for stem cell therapy in humans
Source: Biol Open. 2024 Mar 11;13(3):bio060011. doi: 10.1242/bio.060011 (PMC10924218; doi:10.1242/bio.060011)
Supplement: Supplementary information [file biolopen-13-060011-s1.pdf]

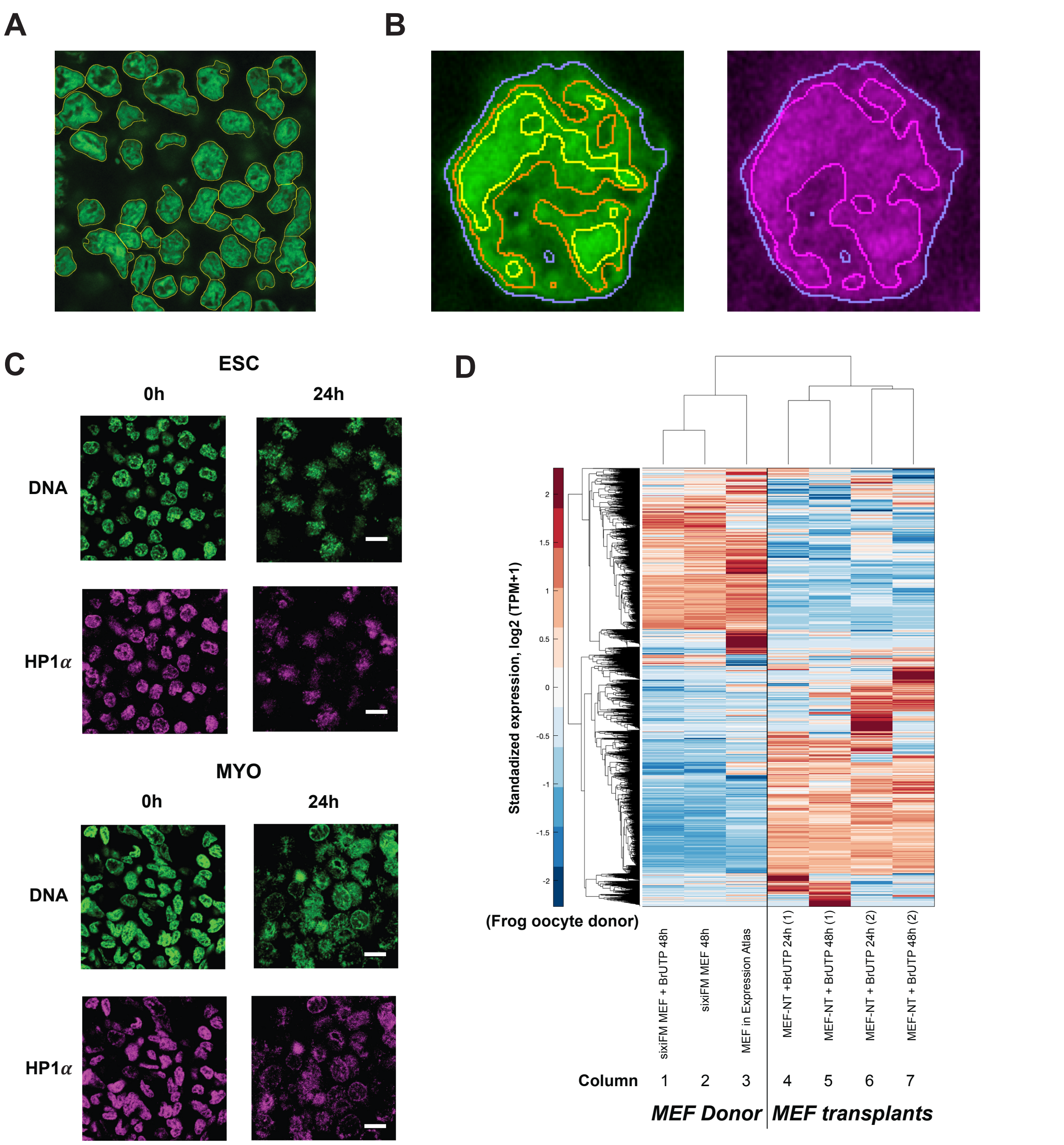

Fig. S1. Chromatin changes and global gene expression shows the induction of a totipotency-like state in MEF by *Xenopus* oocytes is achieved in 24 hours after nuclear transfer.

(A) Image shows the quantification for chromatin area per cell. Yellow lines circle the areas of chromatin (green) for each cell.

(B) Image shows the quantification for areas of chromatin classified to three density levels and the quantification for areas of HP1α. One of injected MEFs in GV of oocyte was magnified to show the details of the classification of chromatin to three density levels. Areas of dense, middle and loose chromatin was circled by yellow lines, between yellow and orange lines, and between orange and purple lines, respectively. Areas of HP1α, overlaid with chromatin of three density levels, was circled by magenta lines.

(C) Images show dispersion of chromatin and loss of HP1α in transplanted ESC and MYO after nuclear transfer. The same experimental procedures were applied as in Fig 1b. Scale bars indicate 10µm.

(D) Heatmap shows that *Xenopus* oocytes reprogram the transcriptional patterns of donor MEF within 24 hours after nuclear transfer (columns 1-3 versus 4-7). For the expression patterns of donor MEF cells, we showed that the expression patters of MEF are almost identical with or without BrUTP pulldown (column 1 versus 2); the expression patterns of the MEF cell line highly resembles the reference transcriptome of primary MEF in Expression Atlas (column 2 versus 3).

Table S1.

Available for download at
https://journals.biologists.com/bio/article-lookup/doi/10.1242/bio.060011#supplementary-data

Table S2.

Available for download at
https://journals.biologists.com/bio/article-lookup/doi/10.1242/bio.060011#supplementary-data

Table S3.

Available for download at
https://journals.biologists.com/bio/article-lookup/doi/10.1242/bio.060011#supplementary-data

Table S4.

Available for download at
https://journals.biologists.com/bio/article-lookup/doi/10.1242/bio.060011#supplementary-data
